# Crystal structure of 4-bromo-5,7-dimeth­oxy-2,3-di­hydro-1*H*-inden-1-one

**DOI:** 10.1107/S2056989024006522

**Published:** 2024-07-19

**Authors:** Sri Hari Galla, Jayalakshmi Sridhar, Joel T. Mague, Xiaodong Zhang, Kira D. White, Qiang Zhang, James P. Donahue

**Affiliations:** a Department of Chemistry Xavier University of Louisiana 1 Drexel Dr New Orleans Louisiana 70125 USA; b Department of Chemistry, Tulane University, 6400 Freret Street, New Orleans, Louisiana 70118-5698, USA; Texas A & M University, USA

**Keywords:** crystal structure, di­hydro­indenone, hydrogen bond, π-stacking

## Abstract

In the title mol­ecule, C_11_H_11_BrO_3_, the di­hydro­indene moiety is essentially planar but with a slight twist in the saturated portion of the five-membered ring. The meth­oxy groups lie close to the above plane. In the crystal, π-stacking inter­actions between six-membered rings form stacks of mol­ecules extending along the *a-*axis direction, which are linked by weak C—H⋯O and C—H⋯Br hydrogen bonds. A Hirshfeld surface analysis was performed showing H⋯H, O⋯H/H⋯O and Br⋯H/H⋯Br contacts make the largest contributions to inter­molecular inter­actions in the crystal.

## Abstract 2

In the title mol­ecule, C_11_H_11_BrO_3_, the di­hydro­indene moiety is essentially planar but with a slight twist in the saturated portion of the five-membered ring. The meth­oxy groups lie close to the above plane. In the crystal, π-stacking inter­actions between six-membered rings form stacks of mol­ecules extending along the *a-*axis directions, which are linked by weak C—H⋯O and C—H⋯Br hydrogen bonds.

## 1. Chemical context

Aberrant expression of protein kinases is a hallmark of several cancers, and small mol­ecules targeting specific kinases are in clinical use as cancer therapeutics (Du & Lovly, 2018 ▸; Kannaiyan & Mahadevan, 2018 ▸; Roskoski, 2023 ▸). Development of resistance to the kinase inhibitors is a frequent occurrence, which motivates a continuing search for new kinase inhibitors (Yang *et al.*, 2022 ▸). One of the key characteristics of the kinase inhibitors is the capacity to form two hydrogen bonds, one as donor and one as acceptor, with the hinge region of the kinase (Arter *et al.*, 2022 ▸; Attwood *et al.*, 2021 ▸). Planarity with two functional groups capable of making the two essential hydrogen bonds, along with other substit­uents to target the unique residues of the ATP binding pocket for potency and specificity, are the fundamental structural features of kinase inhibitors.

We have developed 5-hy­droxy-1,4-naphtho­quinones as HER2 and PIM1 kinase inhibitors (Schroeder *et al.*, 2014 ▸, 2016 ▸; Sridhar *et al.*, 2014 ▸). To circumvent the issue of oxidation-reduction reactions of the quinone moiety, 5,7-dihy­droxy-2,3-di­hydro-1*H*-inden-1-one is currently under development as a new core structure. The new series based upon this platform is capable of making the requisite hydrogen bonds to the kinase hinge region and has potential for functionalization at the 2, 3, 4, 5 and 6 positions to enable specific and potent inhibition of the kinase of inter­est. Bromination serves as an initial step for functionalizing the core structure of 5,7-dimeth­oxy-2,3-di­hydro-1*H*-inden-1-one.

**Figure 1:**
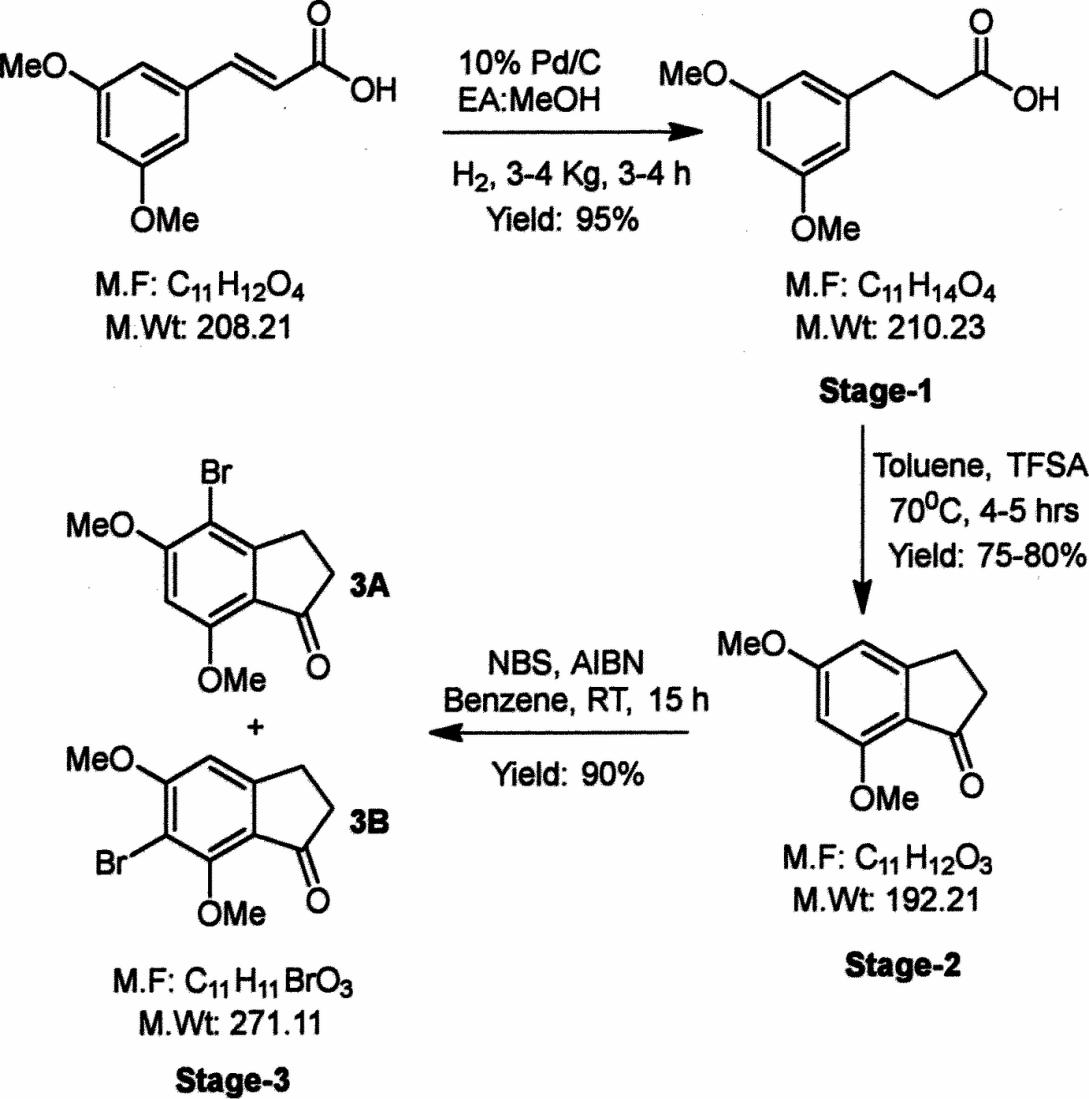
Synthesis scheme for 4-bromo-5,7-dimethoxy-2,3-dihydro-1*H*-inden-1-one.

**Figure 2:**
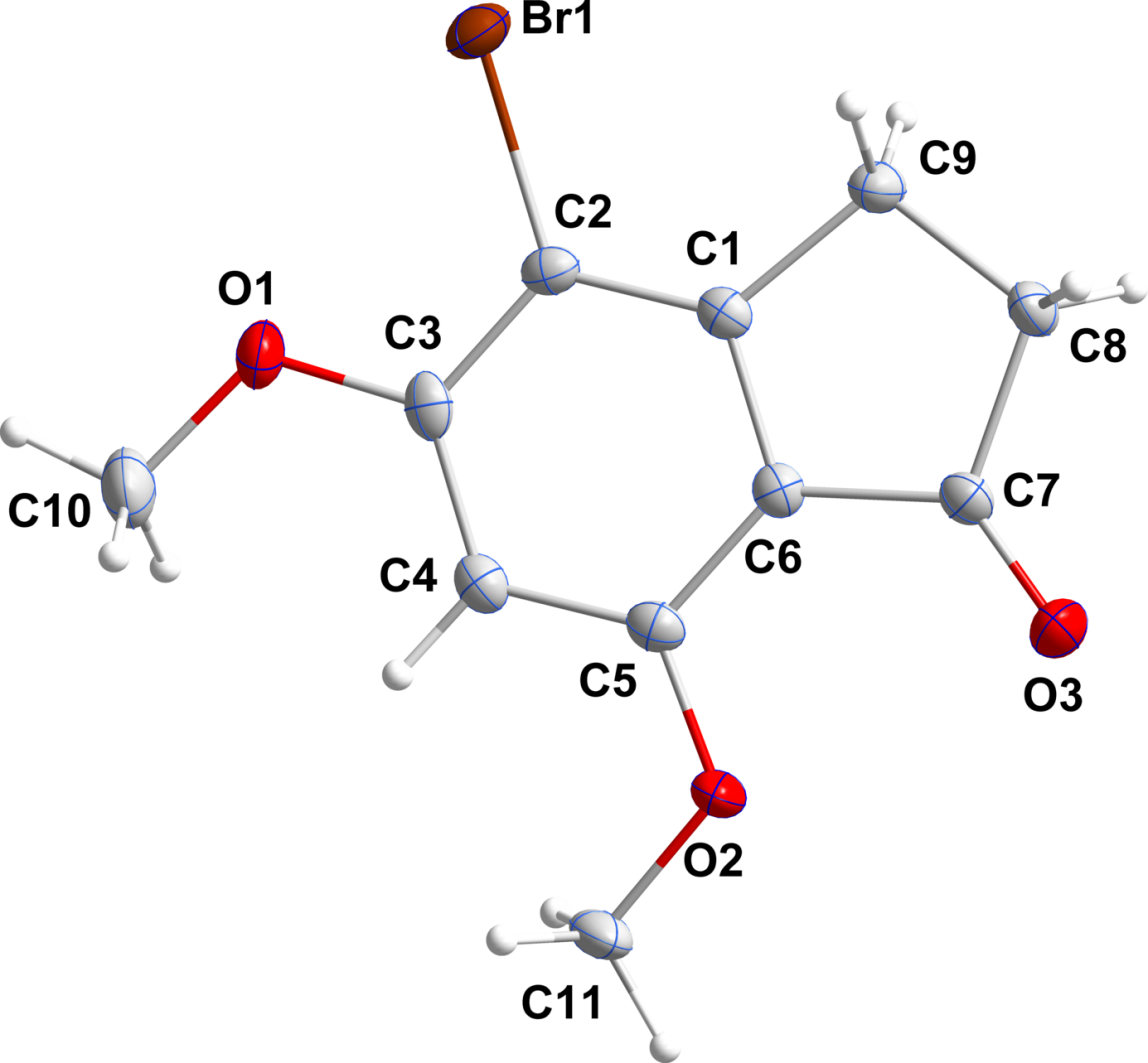
The title mol­ecule with labeling scheme and 50% probability displacement ellipsoids.

Bromination by free radical or electrophilic aromatic substitution mechanisms using *N*-bromo­succinimide (NBS) is known to introduce a bromine atom on an allylic or benzylic carbon atom or on an aromatic ring (Djerassi, 1948 ▸; Li *et al.*, 2014 ▸). When subjected to bromination with NBS in benzene in the presence of azobisisobutyro­nitrile (AIBN) for 15 h at ambient temperature, 5,7-dimeth­oxy-2,3-dihydro-1*H*-inden-1-one yielded a single product. From among the product outcomes depicted as **1A**, **1B** and **1C** in Fig. 1 ▸, NMR spectroscopy indicated that electrophilic aromatic substitution had occurred to form a single species – either **1A** or **1B**. X-ray crystallography has identified the product as **1B** (Fig. 2 ▸), the detailed structural characterization and crystal packing arrangement of which we describe herein.
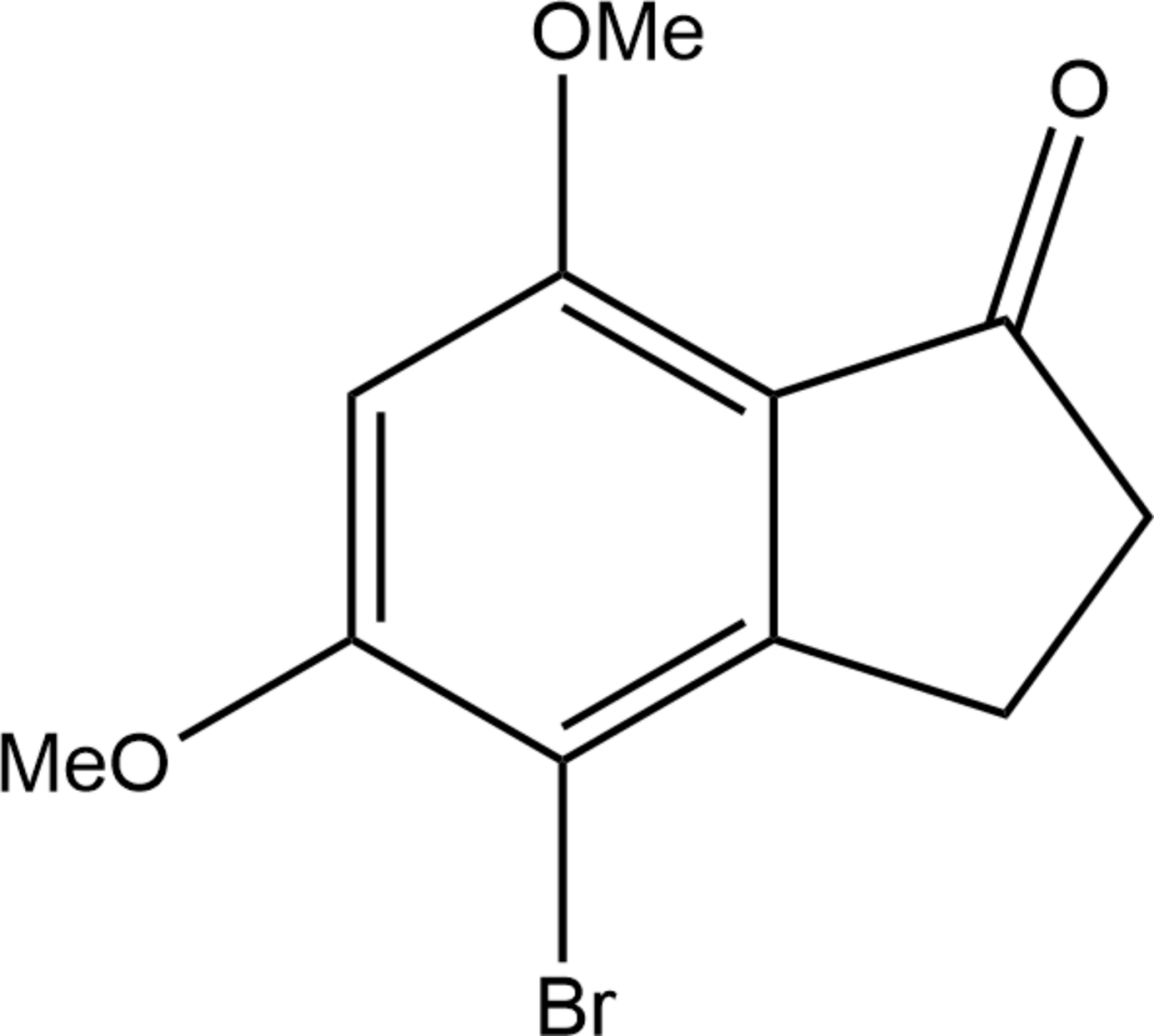


## 2. Structural commentary

The di­hydro­indene moiety is planar to within 0.045 (3) Å (r.m.s. deviation of the contributing atoms = 0.003 Å) with C9 that distance from one side of the mean plane and C8 0.018 (4) Å from the opposite side. This twist in the five-membered ring is towards the upper end of the range seen in related structures (see *Database survey*). Both meth­oxy groups are nearly coplanar with the C1–C6 ring as indicated by the C10—O1—C3—C4 and C11—O2—C5—C4 torsion angles which are, respectively, 2.7 (4) and 6.6 (5)°. All bond distances and inter­bond angles are as expected for the formulation given.

## 3. Supra­molecular features

**Figure 3:**
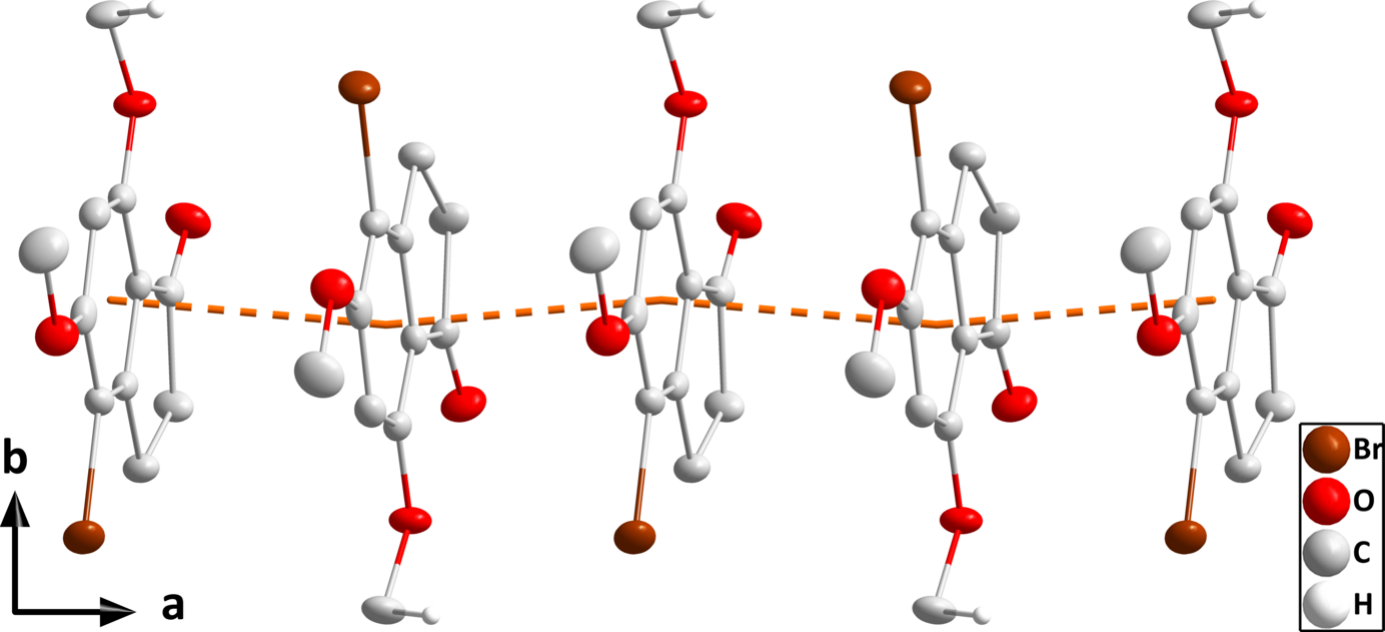
A portion of one stack viewed along the *c*-axis direction with the π-stacking inter­actions depicted by dashed lines. Non-inter­acting hydrogen atoms are omitted for clarity.

**Figure 4:**
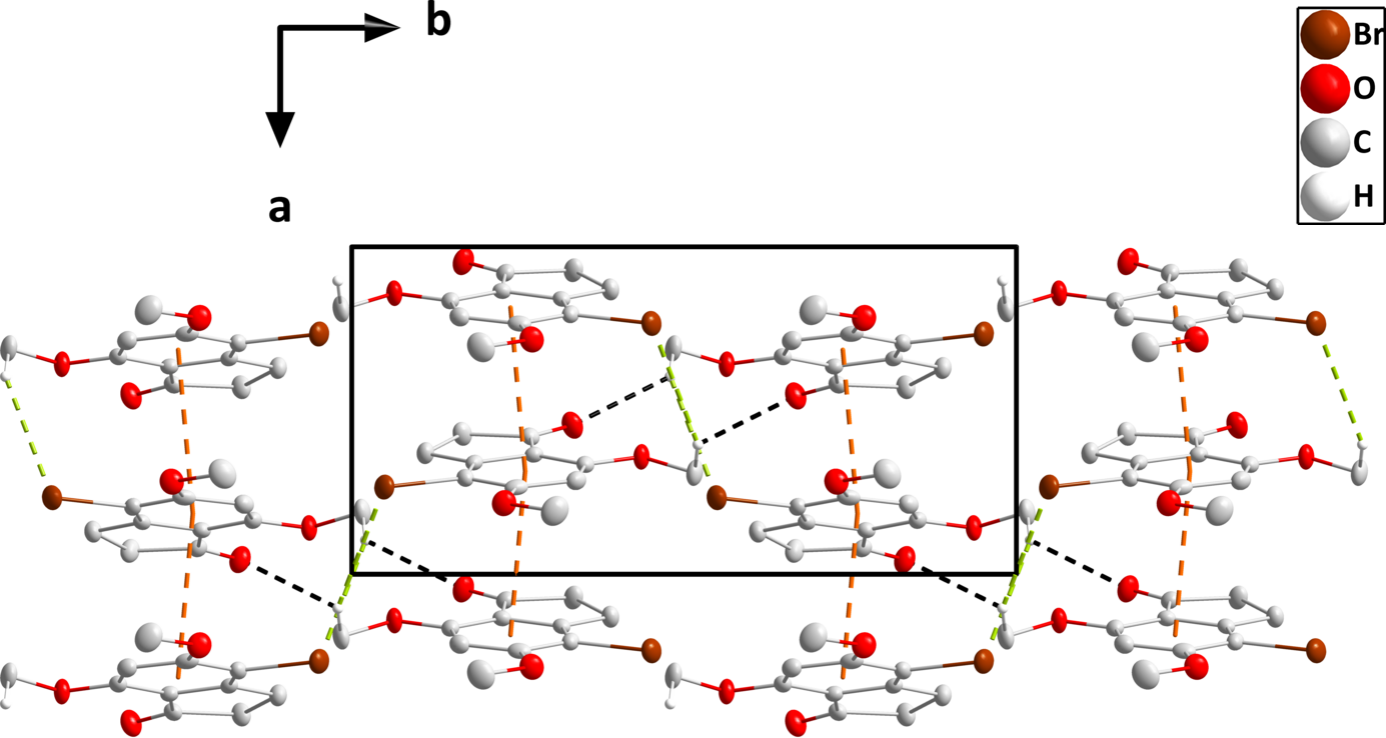
Packing viewed along the *c*-axis direction with the C—H⋯O and C—H⋯Br hydrogen bonds depicted, respectively, by black and green dashed lines. The π-stacking inter­actions are depicted by orange dashed lines and non-inter­acting hydrogen atoms are omitted for clarity.

In the crystal (Fig. 3 ▸), the mol­ecules stack along the *a*-axis direction with significant π inter­actions between the C1–C6 rings [centroid–centroid distance = 3.5606 (16) Å, dihedral angle = 1.61 (13)°, slippage alternates between 0.93 and 0.96 Å]. The stacks are linked by weak C11—H11*C*⋯O3 and C11—H11*C*⋯Br1 hydrogen bonds (Table 1 ▸ and Figs. 4 ▸ and 5 ▸).

## 4. Database survey

**Figure 6:**
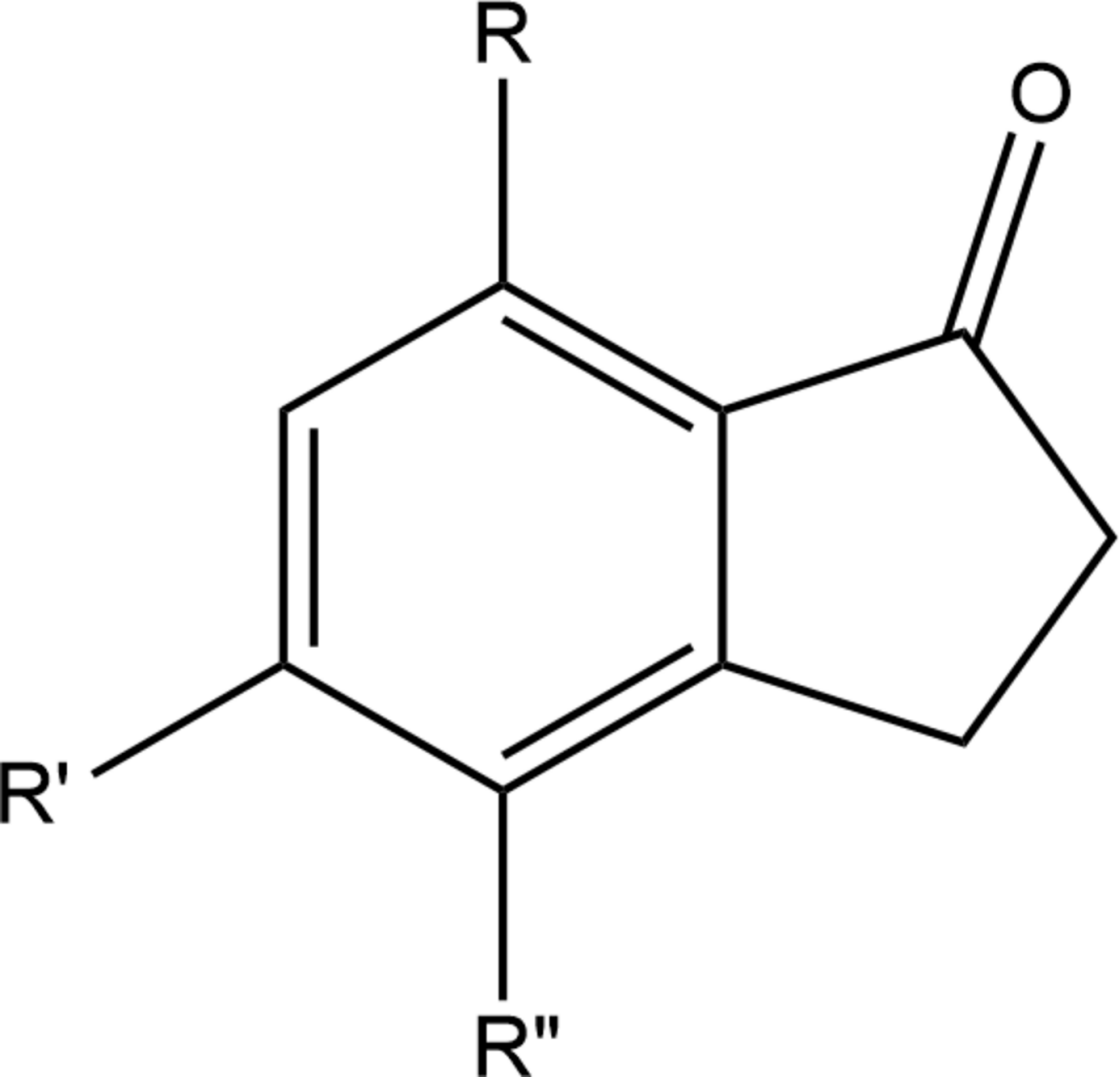
The fragment used in the database survey.

A search of the Cambridge Structural Database (CSD; updated to May 2024; Groom *et al.*, 2016 ▸) with the search fragment shown in Fig. 6 ▸ returned 58 hits of which 25 are most similar to the title mol­ecule with the remainder having additional rings fused to the aromatic ring or being metal complexes. Inter­estingly, there are no examples with three substituents on the six-membered ring, but the search found 18 structures with one substituent, six with two and the unsubstituted parent mol­ecule (*R* = *R*′ = *R*′′ = H, QQQGMJ; Morin *et al.*, 1974 ▸, QQQGMJ01; Peña Ruiz *et al.*, 2004 ▸). There are two structures with *R*′′ = Br, namely AWOBOF (*R* = *R*′ = H; Aldeborgh *et al.*, 2014 ▸) and LAQCAJ (*R* = H, *R*′ = NH_2_; Çelik *et al.*, 2012 ▸), and one with *R* = *R*′ = OMe, *R*" = H (MXINDO10; Gupta *et al.*, 1984 ▸). The other structures with two substituents have *R* = OMe, *R*′ = H, *R*′′ = 4-fluoro­benzoyl (CAPHEJ; Chang & Lee, 2011 ▸), *R* = OH, *R*′ = H, *R*′′ = 4-meth­oxy­benzoyl (CAPHIN; Chang & Lee, 2011 ▸), *R* = H, *R*′ = H, *R*′′ = OPr^*i*^ (CETCAG; Coyanis *et al.*, 2006 ▸) and *R*′ = H, *R* = *R*′′ = Me (MUQCEG; Johnson *et al.*, 2002 ▸). In AWOBOF and LAQCAJ, the C—Br distances are virtually the same as in the title mol­ecule [1.892 (3) Å] and the twist in the five-membered ring is slightly less. Among the other disubstituted mol­ecules, the greatest deviation of the saturated carbon atoms of the five-membered ring from the mean plane of the bicyclic moiety is in CAPHIN [0.091 (2) and −0.122 (2) Å] while the least is in MUQCEG [0.016 (3) and −0.012 (3) Å]. As in the title mol­ecule, the methyl carbon atoms of the meth­oxy groups in CAPHEJ, CETCAG and MXINDO10 lie in or very close to the mean plane of the nine-membered ring system. Where π-stacking of the six-membered aromatic rings occurs in the disubstituted examples, this involves only pairs of mol­ecules (LAQCAJ and MXINDO10) rather than extended stacks.

## 5. Hirshfeld surface analysis

**Figure 7:**
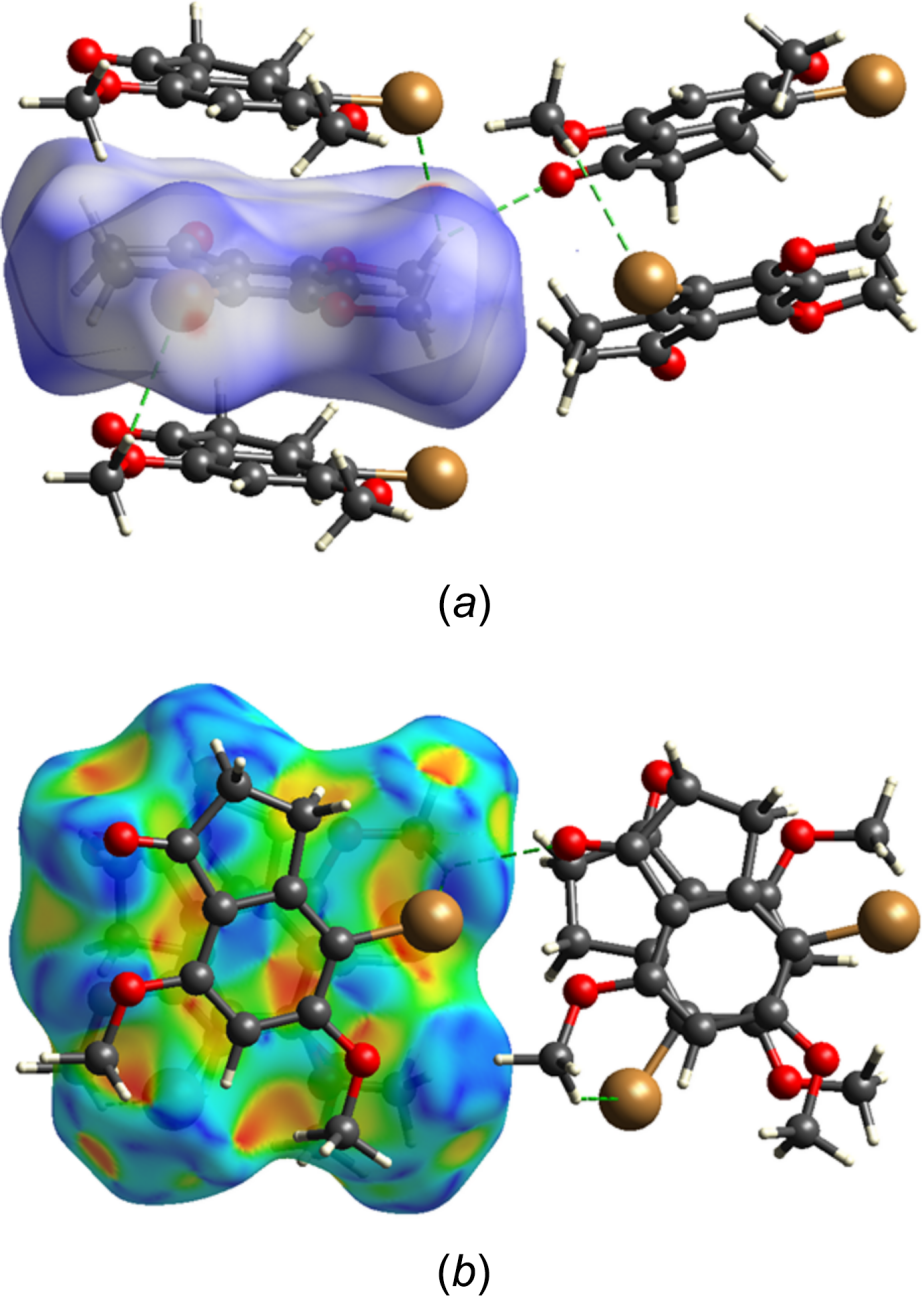
The Hirshfeld surface plotted over (*a*) *d*_norm_ and (*b*) over the shape index including two additional mol­ecules in the stack plus two more in an adjacent stack with the C—H⋯O and C—H⋯Br hydrogen bonds shown by green dashed lines.

**Figure 8:**
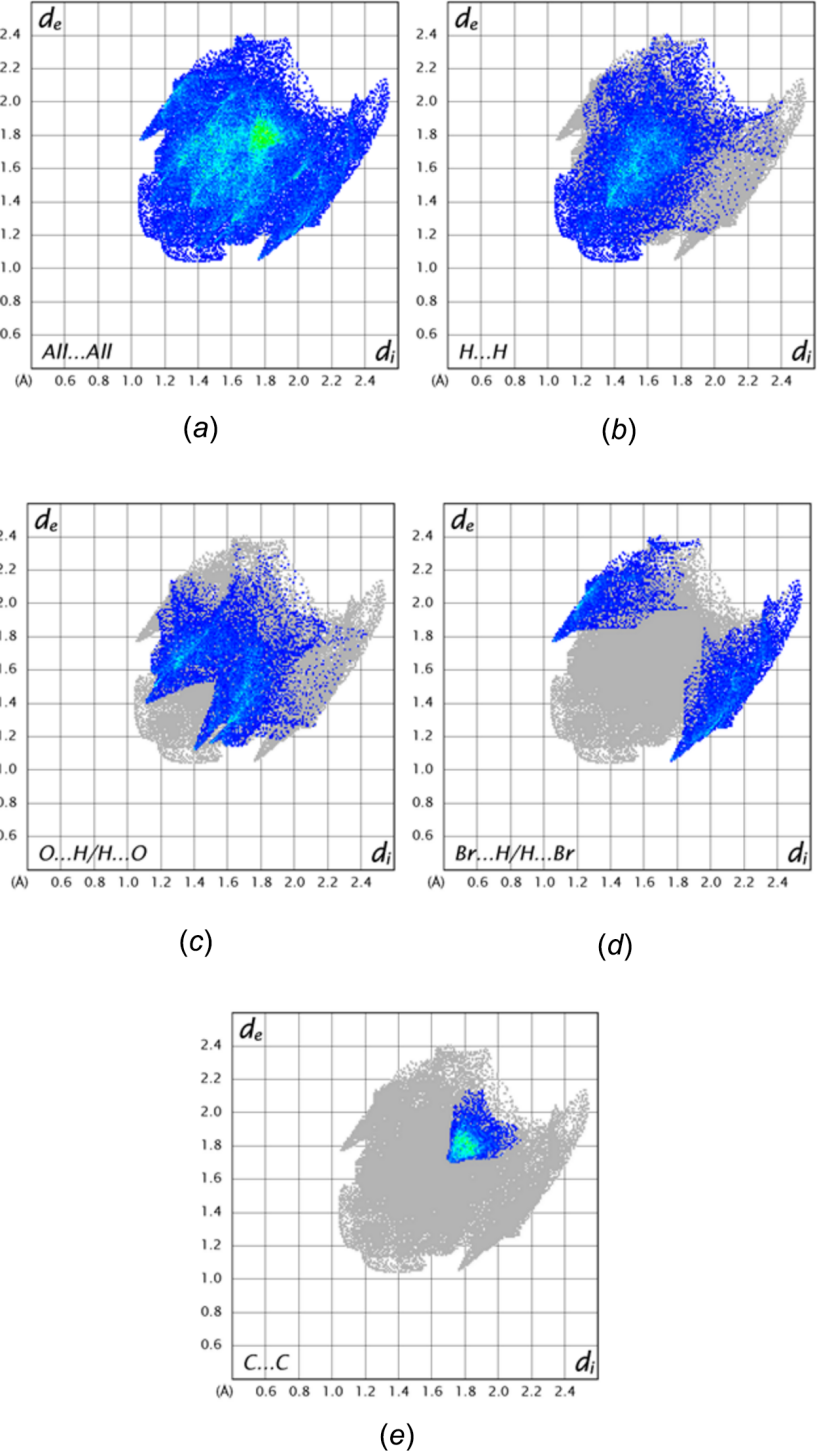
Fingerprint plots showing (*a*) all contacts, (*b*) H⋯H contacts, (*c*) O⋯H/H⋯O contacts, (*d*) Br⋯H/H⋯Br contacts, and (*e*) C⋯C contacts.

The Hirshfeld surface was constructed with *CrystalExplorer 21.5* (Spackman *et al.*, 2021 ▸) with descriptions of the several plots obtained and their inter­pretations described elsewhere (Tan *et al.*, 2019 ▸). Fig. 7 ▸*a* shows the surface plotted over *d*_norm_ in the range −0.1265 to 1.2968 in arbitrary units with four neighboring mol­ecules. The two above and below the surface constitute part of the column formed by the π-stacking inter­actions, while the two at the right are part of an adjacent column showing the C—H⋯O and C—H⋯Br hydrogen bonds that link columns. Fig. 7 ▸*b* shows the surface plotted over the shape function and the flat area in the center containing red and blue triangles clearly shows the π-stacking inter­actions. The 2-D fingerprint plots are shown in Fig. 8 ▸, from which it was determined that H⋯H contacts contribute 37.1% of the total (Fig. 8 ▸*b*) while O⋯H/H⋯O (Fig. 8 ▸*c*) and Br⋯H/H⋯Br (Fig. 8 ▸*d*) contacts contribute, respectively, 26.3% and 16.8%. The C⋯C contacts, which are primarily the π-stacking inter­actions, contribute 9.8%. Other contacts make minimal contributions.

## 6. Synthesis and crystallization

To a solution of 5,7-dimethoxy-2,3-dihydro-1*H*-inden-1-one (1.0 g, 5.2 mmol) in benzene (15 mL) were added *N*-bromo­succinimide (0.93 g, 5.2 mmol) and a catalytic amount of azobisisobutyro­nitrile at room temperature. This reaction mixture was stirred for 15 h, with progress being monitored by TLC. Upon completion of the reaction, the benzene was removed by distillation, and water was added. The resulting slurry was stirred for 30 min, and the crude 4-bromo-5,7-dimethoxy-2,3-dihydro-1*H*-inden-1-one was then collected by vacuum filtration and dried on the filter by continued application of the vacuum for an additional 30 min. Yield: 1.27 g of off-white solid, 4.7 mmol, 90%, m.p. 498–500 K. *R_f_*: 0.4 (1:1 ethyl acetate:hexa­ne). ^1^H NMR (δ, ppm in DMSO-*d*_6_): 6.64 (*s*, 1 H), 3.97 (*s*, 3 H), 3.88 (*s*, 3 H), 2.87–2.84 (*m*, 2 H), 2.55–2.52 (*m*, 2 H). ^13^C NMR (δ, ppm in DMSO-*d*_6_): 201.62, 162.29, 158.85, 157.96, 120.11, 99.75, 96.44, 57.66, 56.55, 37.01, 27.31. HRMS [*M* + H]^+^: ^79^Br calculated, 270.9970; found, 270.9974; ^81^Br calculated, 272.9949; found, 272.9946. The NMR spectra (see supporting information) were acquired using a Bruker 400 MHz spectrometer, while the mass spectrum was obtained using a Thermo LTQ-Orbitrap LC/MS/MS System/UltiMate 3000 HPLC. The compound was crystallized from 15% ethyl acetate in hexane.

## 7. Refinement

Crystal data, data collection and structure refinement details are summarized in Table 2 ▸. Hydrogen atoms were included as riding contributions in idealized positions with isotropic displacement parameters tied to those of the attached atoms. One reflection affected by the beamstop was omitted from the final refinement.

## Supplementary Material

Crystal structure: contains datablock(s) I, global. DOI: 10.1107/S2056989024006522/jy2050sup1.cif

Structure factors: contains datablock(s) I. DOI: 10.1107/S2056989024006522/jy2050Isup2.hkl

Supporting information file. DOI: 10.1107/S2056989024006522/jy2050Isup4.cml

NMR and mass spectra. DOI: 10.1107/S2056989024006522/jy2050sup3.pdf

CCDC reference: 2367432

Additional supporting information:  crystallographic information; 3D view; checkCIF report

## Figures and Tables

**Figure 5:**
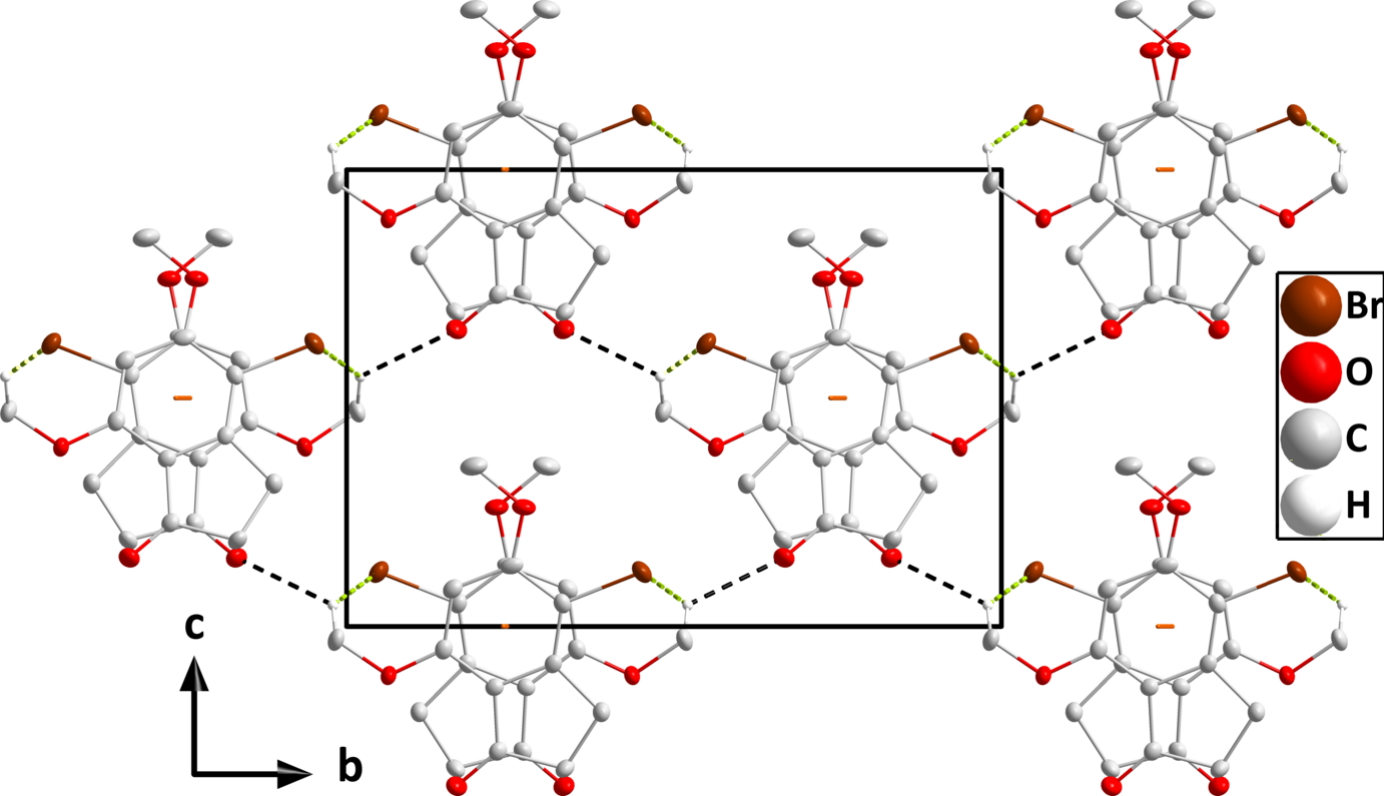
Packing viewed along the *a*-axis direction with the C—H⋯O and C—H⋯Br hydrogen bonds depicted, respectively, by black and green dashed lines. The π-stacking inter­actions are depicted by orange dashed lines and non-inter­acting hydrogen atoms are omitted for clarity.

**Table 1:** Hydrogen-bond geometry (Å, °)

*D*—H⋯*A*	*D*—H	H⋯*A*	*D*⋯*A*	*D*—H⋯*A*
C11—H11*A*⋯Br1^i^	0.98	3.06	3.605 (4)	116
C11—H11*C*⋯Br1^ii^	0.98	2.90	3.683 (3)	137
C11—H11*C*⋯O3^iii^	0.98	2.58	3.288 (4)	129

Symmetry codes: (i) 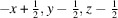; (ii) 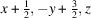; (iii) 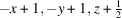.

**Table 2:** Experimental details

Crystal data	
Chemical formula	C_11_H_11_BrO_3_
*M*_r_	271.11
Crystal system, space group	Orthorhombic, *Pna*2_1_
Temperature (K)	150
*a*, *b*, *c* (Å)	7.0928 (5), 14.3933 (10), 10.0419 (7)
*V* (Å^3^)	1025.17 (12)
*Z*	4
Radiation type	Mo *K*α
μ (mm^−1^)	3.99
Crystal size (mm)	0.22 × 0.06 × 0.03

Data collection	
Diffractometer	Bruker D8 QUEST PHOTON 3 diffractometer
Absorption correction	Numerical (*SADABS*; Krause *et al.*, 2015 ▸)
*T*_min_, *T*_max_	0.70, 0.89
No. of measured, independent and observed [*I* > 2σ(*I*)] reflections	13923, 2534, 2366
*R*_int_	0.030
(sin θ/λ)_max_ (Å^−1^)	0.667

Refinement	
*R*[*F*^2^ > 2σ(*F*^2^)], *wR*(*F*^2^), *S*	0.025, 0.056, 1.08
No. of reflections	2534
No. of parameters	139
No. of restraints	1
H-atom treatment	H-atom parameters constrained
Δρ_max_, Δρ_min_ (e Å^−3^)	0.41, −0.39
Absolute structure	Flack *x* determined using 1025 quotients [(*I*^+^)−(*I*^−^)]/[(*I*^+^)+(*I*^−^)] (Parsons *et al.*, 2013 ▸). Refined as an inversion twin
Absolute structure parameter	0.039 (12)

Computer programs: *APEX4* and *SAINT* (Bruker, 2021 ▸), *SHELXT/5* (Sheldrick, 2015*a* ▸), *SHELXL2019/1* (Lübben *et al.*, 2019 ▸; Sheldrick, 2015*b* ▸), *DIAMOND* (Brandenburg & Putz, 2012 ▸) and *SHELXTL* (Sheldrick, 2008 ▸).

## supplementary crystallographic information

### 4-Bromo-5,7-dimethoxy-2,3-dihydro-1H-inden-1-one Crystal data

**Table:**

C_11_H_11_BrO_3_	*D*_x_ = 1.757 Mg m^−^^3^
*M**_r_* = 271.11	Mo *K*α radiation, λ = 0.71073 Å
Orthorhombic, *P**n**a*2_1_	Cell parameters from 9927 reflections
*a* = 7.0928 (5) Å	θ = 2.8–28.2°
*b* = 14.3933 (10) Å	µ = 3.99 mm^−^^1^
*c* = 10.0419 (7) Å	*T* = 150 K
*V* = 1025.17 (12) Å^3^	Block, colourless
*Z* = 4	0.22 × 0.06 × 0.03 mm
*F*(000) = 544	

### 4-Bromo-5,7-dimethoxy-2,3-dihydro-1H-inden-1-one Data collection

**Table:**

Bruker D8 QUEST PHOTON 3 diffractometer	2534 independent reflections
Radiation source: fine-focus sealed tube	2366 reflections with *I* > 2σ(*I*)
Graphite monochromator	*R*_int_ = 0.030
Detector resolution: 7.3910 pixels mm^-1^	θ_max_ = 28.3°, θ_min_ = 2.8°
φ and ω scans	*h* = −9→8
Absorption correction: numerical (*SADABS*; Krause *et al.*, 2015)	*k* = −19→19
*T*_min_ = 0.70, *T*_max_ = 0.89	*l* = −13→13
13923 measured reflections	

### 4-Bromo-5,7-dimethoxy-2,3-dihydro-1H-inden-1-one Refinement

**Table:**

Refinement on *F*^2^	Secondary atom site location: difference Fourier map
Least-squares matrix: full	Hydrogen site location: inferred from neighbouring sites
*R*[*F*^2^ > 2σ(*F*^2^)] = 0.025	H-atom parameters constrained
*wR*(*F*^2^) = 0.056	*w* = 1/[σ^2^(*F*_o_^2^) + (0.0247*P*)^2^ + 0.097*P*] where *P* = (*F*_o_^2^ + 2*F*_c_^2^)/3
*S* = 1.08	(Δ/σ)_max_ = 0.001
2534 reflections	Δρ_max_ = 0.41 e Å^−^^3^
139 parameters	Δρ_min_ = −0.39 e Å^−^^3^
1 restraint	Absolute structure: Flack *x* determined using 1025 quotients [(*I*^+^)-(*I*^-^)]/[(*I*^+^)+(*I*^-^)] (Parsons *et al.*, 2013). Refined as an inversion twin
Primary atom site location: dual	Absolute structure parameter: 0.039 (12)

### 4-Bromo-5,7-dimethoxy-2,3-dihydro-1H-inden-1-one Special details

**Table:**

Experimental. The diffraction data were obtained from 3 sets of frames, each of width 0.50 ° in ω or φ, collected with scan parameters determined by the "strategy" routine in *APEX4*. The scan time was 10.00 sec/frame.
Geometry. All esds (except the esd in the dihedral angle between two l.s. planes) are estimated using the full covariance matrix. The cell esds are taken into account individually in the estimation of esds in distances, angles and torsion angles; correlations between esds in cell parameters are only used when they are defined by crystal symmetry. An approximate (isotropic) treatment of cell esds is used for estimating esds involving l.s. planes.
Refinement. Refinement of F^2^ against ALL reflections. The weighted R-factor wR and goodness of fit S are based on F^2^, conventional R-factors R are based on F, with F set to zero for negative F^2^. The threshold expression of F^2^ > 2sigma(F^2^) is used only for calculating R-factors(gt) etc. and is not relevant to the choice of reflections for refinement. R-factors based on F^2^ are statistically about twice as large as those based on F, and R- factors based on ALL data will be even larger. H-atoms attached to carbon were placed in calculated positions (C—H = 0.95 - 0.99 Å). All were included as riding contributions with isotropic displacement parameters 1.2 - 1.5 times those of the attached atoms. One reflection affected by the beamstop was omitted from the final refinement. Refined as a 2-component inversion twin.

### 4-Bromo-5,7-dimethoxy-2,3-dihydro-1H-inden-1-one Fractional atomic coordinates and isotropic or equivalent isotropic displacement parameters (Å^2^)

**Table:**

	*x*	*y*	*z*	*U*_iso_*/*U*_eq_	
Br1	0.26616 (4)	0.95085 (2)	0.61925 (8)	0.02538 (11)	
O1	0.2173 (3)	0.77072 (17)	0.7610 (2)	0.0259 (5)	
O2	0.3585 (3)	0.56410 (13)	0.3929 (2)	0.0217 (5)	
O3	0.4552 (3)	0.66801 (14)	0.1506 (2)	0.0265 (6)	
C1	0.3410 (4)	0.81569 (19)	0.4184 (3)	0.0153 (6)	
C2	0.2942 (4)	0.8293 (2)	0.5505 (3)	0.0171 (6)	
C3	0.2657 (3)	0.7523 (2)	0.6327 (5)	0.0184 (7)	
C4	0.2862 (4)	0.6621 (2)	0.5822 (3)	0.0191 (7)	
H4	0.266176	0.610159	0.638782	0.023*	
C5	0.3355 (4)	0.6484 (2)	0.4500 (3)	0.0173 (6)	
C6	0.3652 (4)	0.7259 (2)	0.3673 (3)	0.0155 (6)	
C7	0.4207 (4)	0.73116 (19)	0.2271 (3)	0.0172 (6)	
C8	0.4287 (5)	0.8336 (2)	0.1892 (3)	0.0214 (7)	
H8A	0.341666	0.846478	0.114395	0.026*	
H8B	0.558028	0.850884	0.161645	0.026*	
C9	0.3700 (4)	0.8891 (2)	0.3127 (3)	0.0201 (7)	
H9A	0.470112	0.933291	0.339159	0.024*	
H9B	0.252057	0.923961	0.296052	0.024*	
C10	0.1933 (6)	0.6945 (3)	0.8509 (4)	0.0339 (9)	
H10A	0.153765	0.717936	0.938144	0.051*	
H10B	0.312996	0.661020	0.860085	0.051*	
H10C	0.096895	0.652214	0.816037	0.051*	
C11	0.3100 (5)	0.4840 (2)	0.4710 (4)	0.0248 (7)	
H11A	0.320697	0.428012	0.415954	0.037*	
H11B	0.180267	0.490130	0.503313	0.037*	
H11C	0.396091	0.479171	0.547044	0.037*	

### 4-Bromo-5,7-dimethoxy-2,3-dihydro-1H-inden-1-one Atomic displacement parameters (Å^2^)

**Table:**

	*U*^11^	*U*^22^	*U*^33^	*U*^12^	*U*^13^	*U*^23^
Br1	0.03113 (18)	0.02045 (15)	0.02457 (17)	−0.00137 (11)	0.0002 (2)	−0.00798 (19)
O1	0.0341 (14)	0.0296 (14)	0.0140 (12)	−0.0017 (10)	0.0020 (10)	−0.0017 (10)
O2	0.0326 (13)	0.0131 (10)	0.0193 (11)	−0.0007 (9)	0.0028 (10)	0.0019 (9)
O3	0.0366 (13)	0.0231 (11)	0.0200 (14)	0.0059 (9)	0.0048 (9)	−0.0025 (9)
C1	0.0122 (14)	0.0157 (13)	0.0182 (15)	−0.0012 (12)	−0.0021 (11)	0.0019 (11)
C2	0.0160 (15)	0.0153 (14)	0.0201 (16)	0.0006 (12)	−0.0021 (12)	−0.0023 (12)
C3	0.0131 (12)	0.0277 (14)	0.014 (2)	−0.0014 (10)	−0.0025 (13)	0.0032 (17)
C4	0.0184 (15)	0.0200 (15)	0.0188 (17)	−0.0014 (12)	−0.0007 (11)	0.0039 (11)
C5	0.0145 (15)	0.0163 (14)	0.0212 (15)	0.0008 (12)	−0.0018 (12)	0.0017 (12)
C6	0.0128 (14)	0.0173 (14)	0.0163 (15)	−0.0007 (11)	0.0003 (11)	0.0010 (12)
C7	0.0160 (15)	0.0171 (14)	0.0186 (15)	0.0021 (11)	−0.0013 (12)	0.0024 (12)
C8	0.0281 (18)	0.0188 (15)	0.0174 (16)	0.0020 (13)	0.0053 (13)	0.0046 (12)
C9	0.0239 (17)	0.0156 (15)	0.0210 (15)	−0.0010 (12)	0.0022 (13)	0.0001 (12)
C10	0.045 (2)	0.039 (2)	0.0182 (19)	−0.0062 (17)	0.0017 (15)	0.0042 (16)
C11	0.0346 (19)	0.0144 (14)	0.0254 (18)	−0.0033 (14)	−0.0029 (15)	0.0046 (14)

### 4-Bromo-5,7-dimethoxy-2,3-dihydro-1H-inden-1-one Geometric parameters (Å, º)

**Table:**

Br1—C2	1.892 (3)	C6—C7	1.465 (4)
O1—C3	1.359 (6)	C7—C8	1.524 (4)
O1—C10	1.431 (4)	C8—C9	1.532 (4)
O2—C5	1.352 (3)	C8—H8A	0.9900
O2—C11	1.437 (4)	C8—H8B	0.9900
O3—C7	1.215 (3)	C9—H9A	0.9900
C1—C2	1.381 (4)	C9—H9B	0.9900
C1—C6	1.401 (4)	C10—H10A	0.9800
C1—C9	1.512 (4)	C10—H10B	0.9800
C2—C3	1.396 (5)	C10—H10C	0.9800
C3—C4	1.401 (5)	C11—H11A	0.9800
C4—C5	1.387 (4)	C11—H11B	0.9800
C4—H4	0.9500	C11—H11C	0.9800
C5—C6	1.406 (4)		
			
C3—O1—C10	118.6 (3)	C7—C8—H8A	110.3
C5—O2—C11	117.4 (3)	C9—C8—H8A	110.3
C2—C1—C6	120.8 (3)	C7—C8—H8B	110.3
C2—C1—C9	127.5 (3)	C9—C8—H8B	110.3
C6—C1—C9	111.8 (3)	H8A—C8—H8B	108.6
C1—C2—C3	119.4 (3)	C1—C9—C8	104.0 (2)
C1—C2—Br1	120.4 (2)	C1—C9—H9A	111.0
C3—C2—Br1	120.2 (3)	C8—C9—H9A	111.0
O1—C3—C2	116.2 (3)	C1—C9—H9B	111.0
O1—C3—C4	123.4 (3)	C8—C9—H9B	111.0
C2—C3—C4	120.4 (4)	H9A—C9—H9B	109.0
C5—C4—C3	120.3 (3)	O1—C10—H10A	109.5
C5—C4—H4	119.8	O1—C10—H10B	109.5
C3—C4—H4	119.8	H10A—C10—H10B	109.5
O2—C5—C4	124.4 (3)	O1—C10—H10C	109.5
O2—C5—C6	116.3 (3)	H10A—C10—H10C	109.5
C4—C5—C6	119.3 (3)	H10B—C10—H10C	109.5
C1—C6—C5	119.8 (3)	O2—C11—H11A	109.5
C1—C6—C7	109.7 (2)	O2—C11—H11B	109.5
C5—C6—C7	130.5 (3)	H11A—C11—H11B	109.5
O3—C7—C6	128.6 (3)	O2—C11—H11C	109.5
O3—C7—C8	124.0 (3)	H11A—C11—H11C	109.5
C6—C7—C8	107.5 (2)	H11B—C11—H11C	109.5
C7—C8—C9	107.0 (2)		
			
C6—C1—C2—C3	−1.8 (4)	C9—C1—C6—C5	−177.2 (3)
C9—C1—C2—C3	177.4 (3)	C2—C1—C6—C7	−177.9 (3)
C6—C1—C2—Br1	178.9 (2)	C9—C1—C6—C7	2.8 (3)
C9—C1—C2—Br1	−1.9 (4)	O2—C5—C6—C1	178.9 (3)
C10—O1—C3—C2	−177.7 (3)	C4—C5—C6—C1	−1.3 (4)
C10—O1—C3—C4	2.7 (4)	O2—C5—C6—C7	−1.0 (5)
C1—C2—C3—O1	−179.1 (3)	C4—C5—C6—C7	178.8 (3)
Br1—C2—C3—O1	0.3 (3)	C1—C6—C7—O3	179.4 (3)
C1—C2—C3—C4	0.6 (4)	C5—C6—C7—O3	−0.7 (5)
Br1—C2—C3—C4	179.9 (2)	C1—C6—C7—C8	−0.7 (3)
O1—C3—C4—C5	179.8 (3)	C5—C6—C7—C8	179.2 (3)
C2—C3—C4—C5	0.2 (4)	O3—C7—C8—C9	178.4 (3)
C11—O2—C5—C4	6.6 (5)	C6—C7—C8—C9	−1.5 (3)
C11—O2—C5—C6	−173.7 (3)	C2—C1—C9—C8	177.2 (3)
C3—C4—C5—O2	179.9 (3)	C6—C1—C9—C8	−3.6 (3)
C3—C4—C5—C6	0.2 (4)	C7—C8—C9—C1	3.0 (3)
C2—C1—C6—C5	2.1 (4)		

### 4-Bromo-5,7-dimethoxy-2,3-dihydro-1H-inden-1-one Hydrogen-bond geometry (Å, º)

**Table:**

*D*—H···*A*	*D*—H	H···*A*	*D*···*A*	*D*—H···*A*
C11—H11*A*···Br1^i^	0.98	3.06	3.605 (4)	116
C11—H11*C*···Br1^ii^	0.98	2.90	3.683 (3)	137
C11—H11*C*···O3^iii^	0.98	2.58	3.288 (4)	129

Symmetry codes: (i) −*x*+1/2, *y*−1/2, *z*−1/2; (ii) *x*+1/2, −*y*+3/2, *z*; (iii) −*x*+1, −*y*+1, *z*+1/2.
