# Supplementary material for: Crystal structure of 4-bromo-5,7-dimeth­oxy-2,3-di­hydro-1H-inden-1-one
Source: Acta Crystallogr E Crystallogr Commun. 2024 Jul 19;80(Pt 8):873–7. doi: 10.1107/S2056989024006522 (PMC11299745; doi:10.1107/S2056989024006522)
Supplement: Supplementary file 4 [file e-80-00873-sup3.pdf]

Data for Publication

^{1}H-NMR ($CDCl_3$):

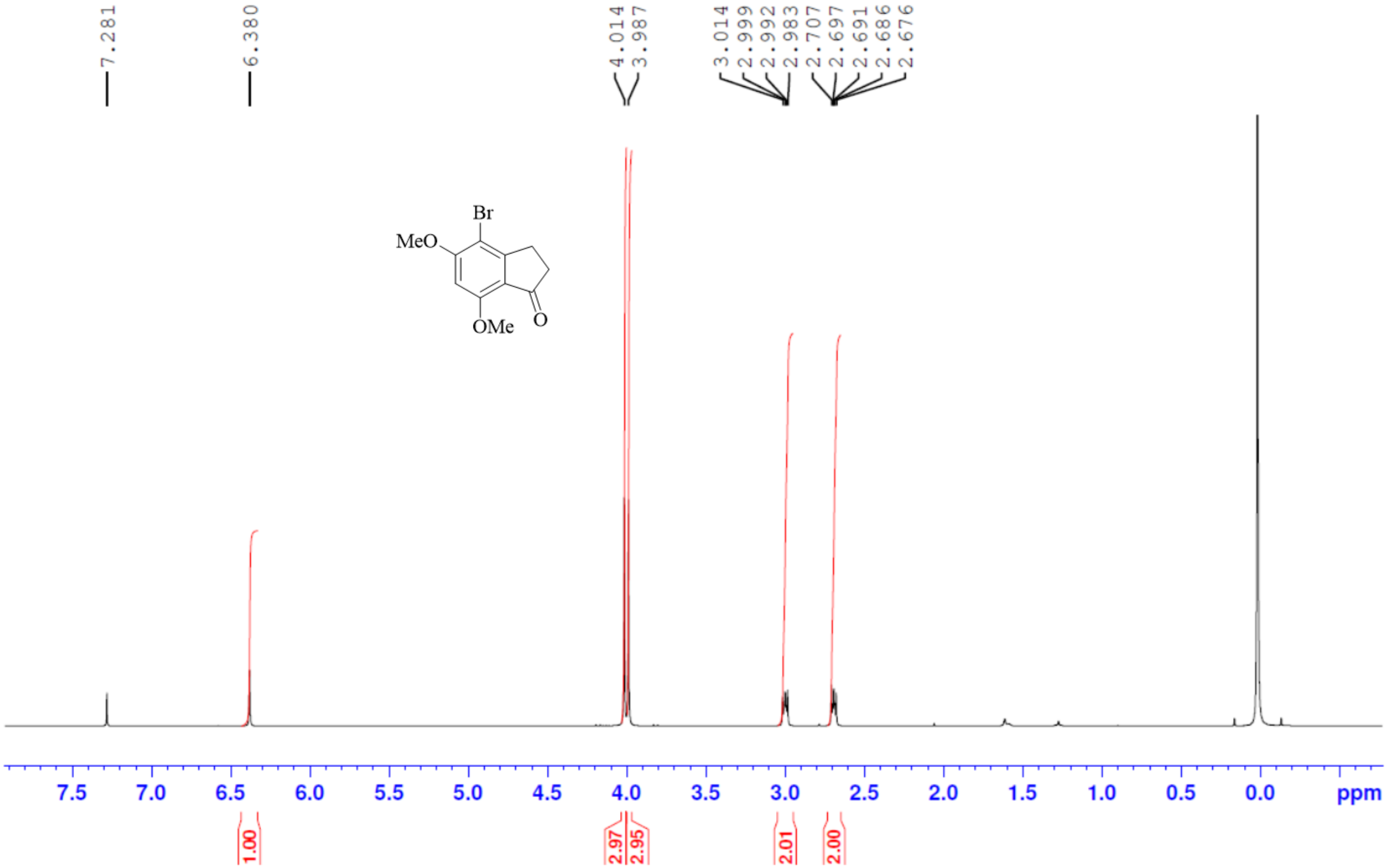

^{13}C-NMR (DMSO-*d*6):

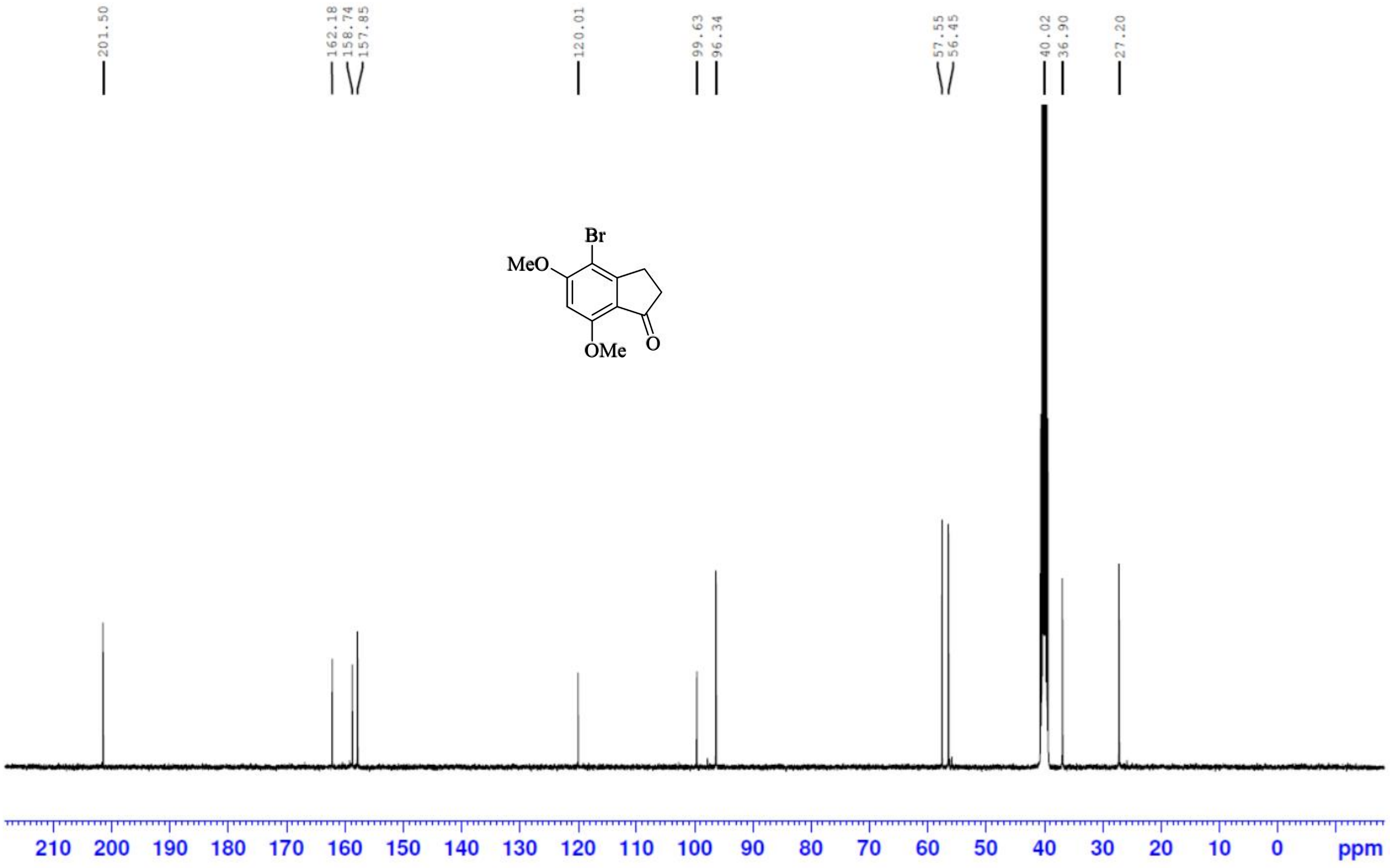

Mol Fomula	M+H	observed
$C_{11}H_{11}BrO_3$	270.9970	270.9974
	272.9949	272.9946

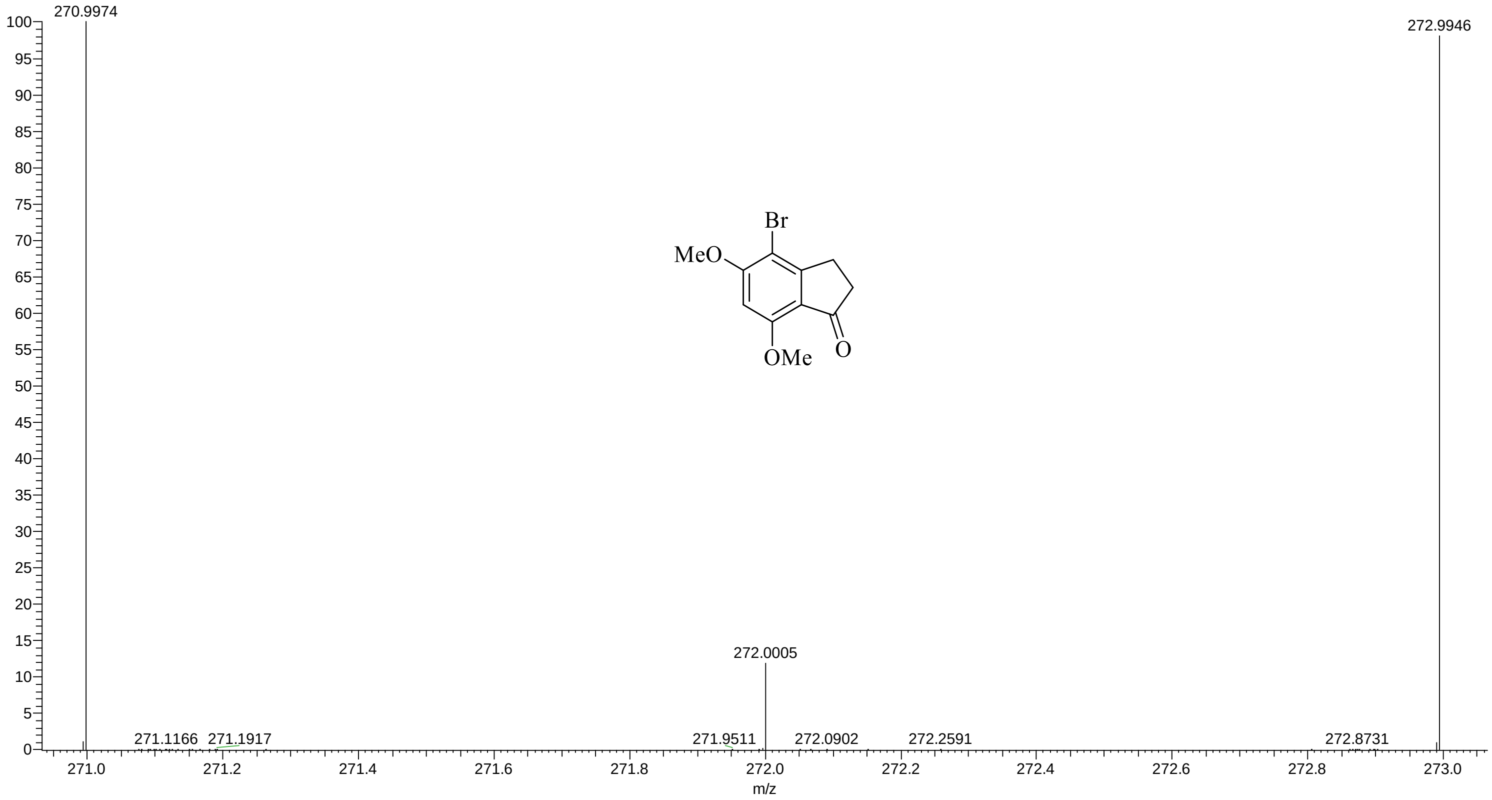

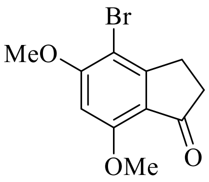

4-bromo-5,7-dimethoxy-2,3-dihydro-1H-inden-1-one(). ^{1}H NMR (400 MHz, $CDCl_3$): δ 6.38 (s, 1H), 4.01 (s, 3H), 3.99 (s, 3H), 3.01-2.98 (m, 2H), 2.71-2.68 (m, 2H). ^{13}C NMR (100 MHz, DMSO-*d6*): δ 201.6, 162.3, 158.8, 157.9, 120.1, 99.7, 96.4, 57.6, 56.5, 37.0, 27.3. HRMS for $C_{11}H_{11}BrO_3$ $[M+H]^+$: Br^{79} calculated: 270.9970; found: 270.9974, Br^{81} calculated: 272.9949; found: 272.9946
